# Methylphenidate promotes the interaction between motor cortex facilitation and attention in healthy adults: A combined study using event‐related potentials and transcranial magnetic stimulation

**DOI:** 10.1002/brb3.1155

**Published:** 2018-11-12

**Authors:** Christoph Berger, Juliane Müller‐Godeffroy, Ivo Marx, Olaf Reis, Johannes Buchmann, Alexander Dück

**Affiliations:** ^1^ Department of Psychiatry, Neurology, Psychosomatics, Psychotherapy in Childhood and Adolescence University Medical Center of Rostock Rostock Germany

**Keywords:** attention, CNV, event‐related potentials, ICF, methylphenidate, motor control, P3, SICF, transcranial magnetic stimulation

## Abstract

**Objective:**

This study investigated simultaneously the impact of methylphenidate (MPH) on the interaction of inhibitory and facilitative pathways in regions processing motor and cognitive functions.

**Method:**

Neural markers of attention and response control (event‐related potentials) and motor cortical excitability (transcranial magnetic stimulation) and their pharmacological modulation by MPH were measured simultaneously in a sample of healthy adults (*n* = 31) performing a cued choice reaction test.

**Results:**

Methylphenidate modulated attentional gating and response preparation processes (increased contingent negative variation) and response inhibition (increased nogo P3). N1, cue‐ and go‐P3 were not affected by MPH. Motor cortex facilitation, measured with long‐interval cortical facilitation, was increased under MPH in the nogo condition and was positively correlated with the P3 amplitude.

**Conclusion:**

Methylphenidate seems particularly to enhance response preparation processes. The MPH‐induced increased motor cortex facilitation during inhibitory task demands was accompanied by increased terminal response inhibition control, probably as a compensatory process.

## 1 INTRODUCTION

The interplay of cognitive, motivational, and motor functions is predominantly controlled by the fronto‐striatal circuit (Seo, Lee, & Averbeck, 2012). The fronto‐striatal circuit is a neuronal network regulating response execution, for example by the selection of actions or by regulating the speed and accuracy of response after a decision was made by the frontal lobe (Forstmann et al., 2010; Lo & Wang, 2006). Beside other neurotransmitters involved in decision making and execution processes, such as GABA or glutamate, adequate dopamine levels are relevant for sufficient functioning of the fronto‐striatal pathways (Baghdadi, Towhidkhah, & Rostami, 2017). A dysregulation of dopamine results in a fronto‐striatal dysfunction which underlies several neurodevelopmental disorders, including attention‐deficit/hyperactivity disorder (ADHD). In ADHD, dopamine affects both inhibitory and excitatory circuits (Bonvicini, Faraone, & Scassellati, 2016). MPH is an indirect dopamine agonist inhibiting the function of the dopamine transporter protein in the cell membrane and in this way increasing the dopamine concentration in the synaptic cleft resulting in a higher signal density at the receptor. MPH was shown to be effective in treating fronto‐striatal dysfunction: MPH has been reported to reduce core symptoms of ADHD in nearly 70% of children with ADHD (for a review, see Wilens, 2008) and a similar efficacy in treating adult ADHD (Castells et al., 2011). For adults and children, however, it was shown that dopaminergic neuronal pathways of the basal ganglia include both a direct “go” path facilitating the execution of an action represented in the cortex and an indirect “nogo” path. For nogo paths, dopamine should inhibit thalamic activity and by that suppress motor actions (Frank, Santamaria, O'Reilly, & Willcutt, 2007). Beside the direct impact of MPH on dopamine availability in the striatum (Volkow, Fowler, Wang, Ding, & Gatley, 2002), MPH is also affecting frontal activity by increasing the concentration of dopamine and norepinephrine in this region (Berridge et al., 2006; Hannestad et al., 2010). It also affects the activity of dopaminergic circuits within the cerebellum (Epstein et al., 2007; Volkow et al., 1998), for a review see the publication of Czerniak et al, 2013 (Czerniak et al., 2013). Taking together, it has been shown that MPH has the potential to modulate both inhibitory and facilitative pathways in regions processing motor and cognitive functions. The impact of MPH on the interaction of these functional different systems remains unclear.

Therefore, it was the aim of our study to investigate the interplay of these neuronal systems as described above, processing cognitive and motor functions in the context of inhibitory and facilitatory task demands.

Such task context demanding adequate motor reactions is the go/nogo test. This task consists of stimulus events requiring an attention focus, either fast responses or the inhibition of inadequate reactions, introduced by the reaction time task pioneer FC Donders in 1860 (Donders, 1969) and is widely used to assess the ability to control impulsive behavior (Castellanos, Sonuga‐Barke, Milham, & Tannock, 2006). Nevertheless recently go/no go task configurations were critically reviewed and described often as suboptimal designed to reliably evoke prepotent motor activity. Therefore, they are reduced in their motor inhibition requirements (Wessel, 2018). Event‐related potentials (ERPs) are an electrophysiological method which allows the examination of sensory and cognitive processes signifying brain responses to various stimuli. Depending on the event category in a cued go/nogo test, ERPs possibly address different attention processes: resource allocation for response preparation (cue condition, contingent negative variation (CNV)), early selective attention processes (N1), response execution (go‐P3) and response inhibition (nogo‐N2 and nogo‐P3). The CNV is a gradient, negative slow ERP to cue stimuli (Walter, Cooper, Aldridge, McCallum, & Winter, 1964), which ceases at the presentation of a target stimulus (Bekker, Kenemans, & Verbaten, 2004). The neuronal source of both CNV and N1 is assumed to be located in the extrastriate cortex (Hillyard & Anllo‐Vento, 1998; Liebrand, Pein, Tzvi, & Kramer, 2017). N2 and P3 differ between go and nogo tasks, with a longer latency and more anterior topography for the nogo‐P3 and a more negatively and frontocentral topography for nogo N2 (Falkenstein, Hoormann, & Hohnsbein, 1999; Spronk, Jonkman, & Kemner, 2008).

Transcranial magnetic stimulation (TMS) is a widely used tool for the evaluation of motor pathways excitability. TMS pulses are delivered to the primary motor cortex. Motor‐evoked potentials (MEPs) are measured at surface electrodes, usually placed on small hand muscles, such as the first dorsal interosseus muscle (FDI). Paired‐pulse TMS protocols lead to either inhibitory or facilitatory effects on the MEP depending on the interstimulus interval (ISI) between the conditioning and the test pulse and on the intensity of both stimuli (Kujirai et al., 1993; Valls‐Sole, Pascual‐Leone, Wassermann, & Hallett, 1992). Four different kinds of ISIs are described in the literature. An ISI of 2 to 5 ms decreases the MEP, which is usually called short‐interval cortical inhibition (SICI). An ISI of 6–25 ms causes facilitatory effects on the MEP, called intracortical facilitation (ICF). A second facilitation period of around 50 ms is called long‐interval cortical facilitation (LICF). Longer periods of around 100 ms ISI induce another inhibition effect, which is called long‐interval cortical inhibition (LICI).

Research using TMS paradigms to analyze motor cortex excitability has been initially conducted during resting state, but increasingly also under facilitatory or inhibitory task conditions. Several studies have shown a strong modulation of corticospinal excitability in go/nogo tasks in order to suppress or facilitate go responses (Kinoshita, Yahagi, & Kasai, 2007; Kratz et al., 2009; Nakata et al., 2006; Sohn, Dang, & Hallett, 2003; Yamanaka et al., 2002). A recent review discusses theoretical models of movement regulation with the focus on TMS as a physiological marker of motor inhibition during processes of action stopping and action preparation (Duque, Greenhouse, Labruna, & Ivry, 2017). The authors describe a different expansion of inhibitory processes in the motor system, reaching from focal to a broad phenomenon, which are also evident in task‐irrelevant muscles, especially when the ongoing action has to be rapidly aborted. In another study on motor cortex excitability and its modulation by attention in healthy adults, it was demonstrated that SICI, measured by TMS, decreases under task conditions with attention focus on an internal or external locus, compared to a resting condition (Ruge, Muggleton, Hoad, Caronni, & Rothwell, 2014). The authors suggested that the disturbation of SICI which has been found in disorders like Tourette's syndrome (Orth & Rothwell, 2009), first‐episode schizophrenia (Wobrock et al., 2008), or ADHD (Gilbert, Isaacs, Augusta, Macneil, & Mostofsky, 2011; Moll, Heinrich, Trott, Wirth, & Rothenberger, 2000) may not only being explained by impaired intracortical GABA circuits per se. They provided an additional interpretation saying that motor cortical excitability might be modulated by different cognitive (attentional) states associated with disorders named above.

Beside the use of MPH in the treatment of ADHD (Wilens, 2008), there is evidence for manifold cognitive effects of MPH in the general population. MPH affects working memory, processing speed, verbal learning, attention, and vigilance (Linssen, Sambeth, Vuurman, & Riedel, 2014). That was proven by a study wherein a go/nogo task was performed by healthy adults and ERP and TMS measures were taken (Hoegl et al., 2011). For ERP, authors found an increased response evaluation indexed by an elevated P3 under MPH, but only for go trials, and not for nogo trials.

Many studies have shown that the P3 ERP component is reduced in adult and juvenile subjects suffering from ADHD in both auditory and visual modality (Barry, Johnstone, & Clarke, 2003). A medication with MPH seems to normalize the P3 activity at all ages (Groom et al., 2010; Klorman, Salzman, Pass, Borgstedt, & Dainer, 1979; Sunohara et al., 1999; Verbaten et al., 1994). Broyd et al. investigated MPH effects on the performance and the ERP of children with ADHD in a go/nogo task (Broyd et al., 2005). In this study, N1 and P2 amplitudes were found to be leveled while commission errors were normalized after MPH medication, the latter suggesting improved response inhibition.

Partially divergent results were found in a couple of studies with adult patients suffering from ADHD using a stop signal task (Ohlmeier et al., 2007; Overtoom et al., 2009). No effect of MPH was found for low doses of MPH (0.4 mg/kg) on any of the ERP. Under high doses of MPH (0.6 mg/kg), however, N1 was increased while P3 was decreased, with no effects on reaction time under the go condition (Overtoom et al., 2009).

Methylphenidate has been shown to influence motor cortex excitability in both inhibitory and excitatory neuronal circuits in normal adults (Gilbert et al., 2006; Kratz et al., 2009). With children as well as with adults, MPH is extensively used in the treatment of ADHD. The substance is known to restore disturbed cortical motor inhibition and facilitation in children (Buchmann et al., 2007; Gilbert et al., 2006; Moll et al., 2000) and to improve motor disinhibition in adults (Schneider et al., 2011). Remarkably, contradictory results have been obtained for SICI and ICF in healthy adults after the ingestion of a single dose of MPH (Gilbert et al., 2006; Ilic, Korchounov, & Ziemann, 2003; Kirschner et al., 2003; Moll, Heinrich, & Rothenberger, 2003). But again, it still remains unclear whether modulations in motor cortical facilitation are resulting only by the direct effects of MPH on the striatal dopaminergic pathways. It has been reported that changes in motor cortex excitability can be induced also by attentional states (Conte et al., 2007; Rosenkranz & Rothwell, 2004; Ruge et al., 2014; Thomson, Garry, & Summers, 2008). Possibly, changes in the motoric system in experiments with MPH application at least partly depend on the MPH‐induced modulations of frontal regions processing attentional states.

To follow‐up upon this hypothesis, simultaneous measurement of ERP and TMS parameters provides a useful technique to analyze the interaction between attention processes and motor cortex excitability. This combined method was first used in a study on adults analyzing corticospinal excitability with a single pulse TMS over the left primary motor cortex and frontocentral ERP measurement for two types of go/nogo tasks with different movement instructions (push‐go and release‐go; Yamanaka et al., 2002). TMS results speak for a task‐dependent modulation of corticospinal excitability, but ERP measurements, however, were not different between the two tasks.

Hoegl et al. investigated effects of MPH on processes of response inhibition using paired‐pulse TMS (SICI) and ERP measurement in healthy adults performing a go/nogo task (Hoegl et al., 2011). Their regression analysis revealed that an increased inhibition evaluation process, indexed by nogo‐P3, was associated with a decreased motor cortex inhibition measured by short intra cortical inhibition (SICI). This effect was only present for the MPH condition. The authors interpreted this result as a physiological need for a higher terminal response control and related resource allocation in case of lower inhibitory effects in the motor system.

In a more recent study, the same team used a similar paradigm with children diagnosed with ADHD (Heinrich, Hoegl, Moll, & Kratz, 2014). ADHD patients differed from the control group in the associations between performance, ERP, and TMS measures. The authors reported for ADHD patients but not for healthy controls an association between an increased inhibition evaluation process (indexed by nogo‐P3) and an increased motor cortex inhibition after inhibiting the response (SICI at 500 ms post stimulus). From these results, a deviant neural implementation of motor control in children with ADHD was inferred, possibly reflecting compensatory cognitive mechanisms of reduced activation in inhibitory intracortical interneurons within the motor cortex.

While these prior studies focused on response inhibition processes and analyzed TMS measure of inhibitory motor cortex activity, we aimed at widening the view on TMS measures also analyzing the relation between facilitatory motor cortex processes and attentional response control. It has been shown that MPH has the ability to modulate cognitive and motoric functions by increasing catecholamine (dopamine and noradrenaline) availability in the fronto‐striatal pathways, but MPH effects on the cognitive–motoric interactions remain unclear. In this study, our group therefore investigated the effects of MPH on the potential associations between ERP indexing cognitive processes and various TMS measurements indexing motor‐cortical excitation in a go/nogo choice reaction task at the same time. In a previous publication, the authors reported the effects of MPH on the motor cortex excitability in terms of motor‐evoked potentials (MEP) as responses to TMS pulses under different task conditions. They found stronger effects of MPH on facilitatory processes compared to effects on intracortical inhibition (Buchmann et al., 2010). The data presented here were analyzed for effects of MPH on the attention processes indexed by ERPs and alterations in the interplay of these attention processes with motor cortex excitability possibly induced by MPH.

## 2 MATERIAL AND METHODS

### 2.1 Subjects

Thirty‐one healthy adults (14 female, 17 male) volunteering for the study were recruited from medical staff and students. The mean age was 28, ranging from 18 up to 43 years. Subjects were tested extensively: neurological examination, structured clinical interview I and II for psychiatric disorders [German version, SCID I + II (Maffei et al., 1997; Spitzer, Williams, Gibbon, & First, 1992; Williams et al., 1992)], Conners Adult ADHD Rating Scale (CAARS, ADHD Index <PR 84), Wender Utah Rating Scale (WURS, overall score < 30), intelligence (Hamburg–Wechsler Intelligence Test, IQ > 85), and concentration tests [attention performance test d2 (Brickenkamp, 2002)]. Criteria for an exclusion from the study were a history of neurological or severe medical disorder, head or spinal cord trauma, epileptic seizures, brain lesions or neurosurgery, any cardiac pacemakers or pregnancy. Furthermore, volunteers with major psychiatric disorders such as dementia, major depression, bipolar affective, psychotic, obsessive‐compulsive, anxiety and addiction disorders (DSM‐IV‐SCID I) and personality disorders (SCID II) were excluded. Two persons were excluded because they suffered from ADHD according to the criteria from CAARS and WURS; six were excluded by at least one of the criteria listed above. Subjects included received a reward as book or cinema vouchers (30 €). The experiment was performed in accordance with the declaration of Helsinki and approved by the local University Ethics Committee. A written informed consent was obtained from each participant. The permission of the local authority (German Drug Administration, Bundesarzneimittelinstitut, and Bundesopiumstelle) was given.

### 2.2 MPH application and titration

Extensive instructions were given to all subjects. Volunteers received a start dose of 10 mg MPH. Doses were increased stepwise by 10 mg/week being adjusted for body weight during the last week, until the final end dose of 1 mg/kg body weight or 60 mg MPH maximal was reached. Side effects mostly regarded loss of appetite, headache, and subjectively experienced tachycardia (“feel my heart beat”). Occasionally, subjects reported sleep disturbances. During the titration period, no dropouts from the study occurred.

Methylphenidate level was measured by LC‐MS (fluid chromatography with mass‐spectrometric evaluation by means of a quadropoldetector in high vacuum). MPH clearance was calculated by the formula: dose rate × distribution volume.

### 2.3 Transcranial magnetic stimulation

All volunteers wore bathing caps (put on above the EEG caps) enabling investigators to mark the point of optimal excitability (POE as described below) with an ink pen. Left motor cortex stimulation was performed over the hand area with a focal 8‐shaped coil (outside diameter of one half‐coil 7 cm) connected to a MagPro Option device (Denmark). Elicited electromyographic responses were recorded from the contralateral first dorsal interosseus muscle (FDI) using surface electrodes with a belly‐tendon montage.

The POE of the cortical representation of contralateral FDI was determined by inching the coil over the area of the motor cortex, stimulating with an intensity of approximately 5%–10% above resting motor threshold (RMT). RMT of the right FDI muscle was defined as the lowest stimulation intensity which produced an EMG response of at least 50 μV measured peak‐to‐peak in at least six out of ten stimuli (Rossini et al., 1994). The “1 mV threshold” (1 mVT) of right FDI muscle contralateral to TMS was determined as the stimulation intensity which produced a MEP with an amplitude between 0.5 and 1.5 mV measured peak‐to‐peak in at least eight out of ten stimuli.

Short‐interval cortical inhibition investigation was performed with an ISI of 3 ms (ISI 3) and a stimulator output of 90% RMT for the conditioning stimulus and 1 mVT stimulation intensity for the test stimulus (Kujirai et al., 1993). ICF investigation was performed with an ISI of 13 ms (ISI 13). An additional investigation of facilitatory effects (LICF) was performed under the condition of an ISI of 50 ms (ISI 50), both stimuli applied with 1 mVT intensity (Valls‐Sole et al., 1992). LICI was established using ISIs of 100 ms (ISI 100), both stimuli with 1 mVT intensity (Valls‐Sole et al., 1992).

### 2.4 Go/nogo task and TMS conditions

All volunteers performed two experimental sessions, a baseline session with no MPH and after 6 weeks a session with the final end dose of MPH administration. Each session consists of 5 blocks of a visual cued stimulus‐reaction task (cued go/nogo task): yellow light =attention cue followed after 1,600 ms by one of two targets, a red after yellow light =go and green following yellow light =nogo. Subjects were asked to press a button with the thumb of the right hand as quickly as possible when the combination “red light following a yellow one”= go occurred in the sequence of visual stimuli. A total of 30 cue‐target pairs of each task condition were offered pseudo randomly per task session within a cue‐to‐cue interval of 4,800 ± 1,600 ms.

**Figure 1:**
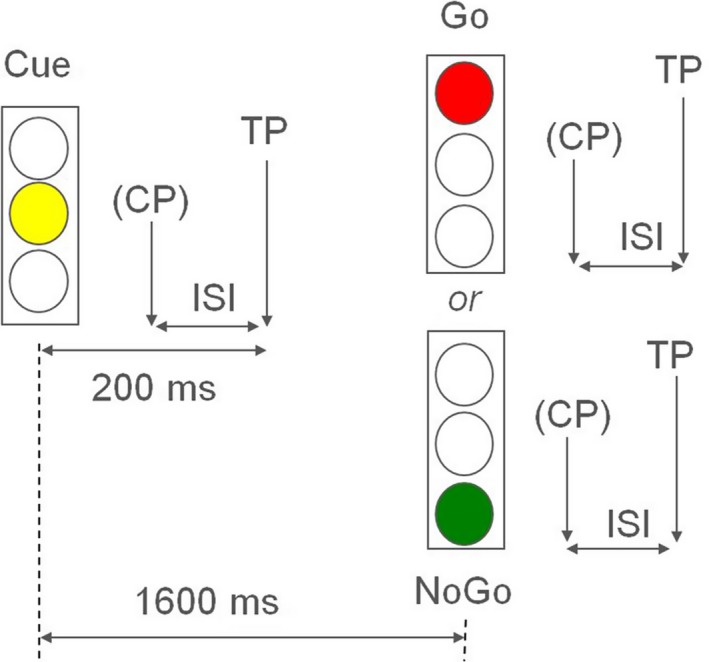
Go/no go paradigm. (CP) = conditioning TMS pulse, or not applied in single pulse paradigm, TP, target pulse; ISI, inter stimulus interval in paired‐pulse paradigms 3, 13, 50, 100 ms

Transcranial magnetic stimulation pulses were triggered in a fixed interval of 200 ms after the cue, go, or nogo stimulus. For the paired‐pulse paradigms, this 200 ms interval was locked to the target TMS pulse and the preceding conditioning TMS pulse was closer to the task condition stimulus (see Figure 1).

Each go/nogo task block was separately accompanied by one of the 5 TMS protocols (single pulse, ISI 3, 13, 50, 100 ms). Single or paired TMS pulses were carried out for the cue condition as well as for the go and nogo condition, respectively in 15 of the 30 trials of each condition. This procedure was performed in every task block with at least 5 s time elapsed between the TMS pulses.

By proceeding the described way, five different MEP amplitudes were obtained for each task condition: MEP responses to the one single and four paired‐pulse protocols under task condition, respectively.

The five sequences of go/nogo task with different pulse protocols of single and paired pulse were counterbalanced in pseudo‐randomized order across all persons. At all times, TMS procedure was performed in the mid‐morning and at least 3 hr after the intake of MPH.

### 2.5 Brain electrical activity and event‐related potentials

The electroencephalogram (EEG) was recorded with sintered Ag/AgCl electrodes and an abrasive, hypertonic electrolyte (© everi, Spes Medica, Italy) from 17 sites according to 10/20 system (Fp1, FP2, F7, F8, C3, C4, T3, T4, T5, T6, P3, P4, O1, O2, Fz, Cz, Pz, recording reference: FCz, ground electrode: CPz). Sampling rate was set to 2,500 Hz and recording filter bandwidth was set to 0.02–1,000 Hz. Impedance was kept below 10 kΩ. Recording system was BrainAmp (BrainProducts, Gilching, Germany).

Offline EEG was preprocessed with BrainVision Analyzer (BrainProducts). The data were first filtered with a Butterworth filter, 48 dB/Oct, bandwidth 0.3–50 Hz, including a 50 Hz‐Notch filter. Trial data from all TMS protocol sessions were segmented out from −100 to 800 ms in relation to cue, go, and nogo marker for further processing. As interaction of TMS pulse application with performance and ERP measures should be avoided, trials with TMS pulses were excluded from this segmentation, resulting in 75 trials for the go and nogo condition and 150 trials for the cue condition. After a first visual inspection excluding trials with strong movement artifacts, an independent component analysis (ICA) was used to eliminate artifacts due to eye movement, temporal electrode noise, and cardioballistic activity. After ICA, data were again visually inspected for residual artifacts. The EEG data were re‐referenced to a common reference obtained by averaging all channels. Trials with amplitudes >100 µV as well as with omission and false alarm errors were excluded from final averaging over the same task condition (attention, go, and nogo). ERP scoring for CNV, N1, N2, and P3 was computed as the mean amplitude for specific intervals and locations selected by visual inspection of the grand mean ERP averages of the EEG data and further in accordance with prior studies (Heinrich et al., 2014; Hoegl et al., 2011): N1 from 60 to 110 ms, N2 from 170 to 280 ms, and P3 from 250 to 400 ms; N1 at Fz and N2, P3 either at Cz after nogo signal or at Pz after cue or go signal; CNV at FZ from 100 to 0 ms before go or nogo signal.

### 2.6 Statistical analysis

We analyzed the effects of MPH medication on ERP amplitudes with repeated measures ANOVA, separately for the ERP N1, N2, and P3 as dependent variables. Medication with MPH (baseline vs. full medication) and task condition (go, nogo, cue) were modeled as independent factors. Greenhouse‐Geisser correction was used, when the assumption of sphericity was not met for the inner subject factor task condition. In case of significant interaction effects for task condition and MPH medication or significant main effects for at least one task condition, post hoc *t* tests were calculated to determine significant differences between the ERP at factor levels. The effects of MPH on CNV and reaction time were analyzed by using paired *t* tests; the effect of MPH on hit rate (not normally distributed) was analyzed with Wilcoxon test.

In addition to the modulatory MPH effects on the TMS MEP responses, including single pulse MEPs reported earlier (Buchmann et al., 2010), we analyzed the effect of MPH on the relative change of MEP response to paired TMS pulse protocols in relation to the single pulse MEP response (SICI, LICI, ICF, LICF in %), this way directly addressing the inhibitory or facilitatory impact of the paired‐pulse TMS protocols. These TMS effects were not normally distributed; therefore, we used Friedman tests to analyze the main effect of task condition and Wilcoxon tests to analyze the main effect of MPH and the interaction of both experimental factors.

Pearson's correlations were calculated to determine the relation between MPH serum level, MPH clearance and TMS effects, task performance or ERP when these experimental measures were significantly different under MPH medication compared to baseline. Bonferroni correction with a threshold of 0.02 (0.05/2: MPH serum level and clearance) was imposed on the correlation analysis to avoid alpha error accumulation for multiple testing.

We aimed further to analyze the association between motor cortex excitability measured by TMS and cognitive dimensions of response control measured by ERP components and how these associations are affected by MPH treatment. Therefore, we performed Spearman's correlations between TMS measures (SICI, LICI, ICF, LICF in %), hit rate, reaction time and ERP (N1, N2, P3, CNV) separately for each task condition (rt and hit rate only for go condition, CNV only for attention) and separately for ERP and TMS measurements with and without MPH medication.

Again, a strict Bonferroni correction with a threshold of 0.0042 (0.05/12:4 TMS measures, 3 task conditions) was imposed on all ERP correlation analyses in order to level the alpha error accumulation for multiple testing.

Additionally, we tested for possible gender differences in MPH effects on the TMS, ERP, and performance measures modeling gender as a between‐subject factor within repeated measures ANOVAs or Mann–Whitney *U* tests, depending on the statistical distribution of the depending variables.

## 3 RESULTS

### 3.1 Effects of MPH medication on TMS excitatory effects

**Figure 2:**
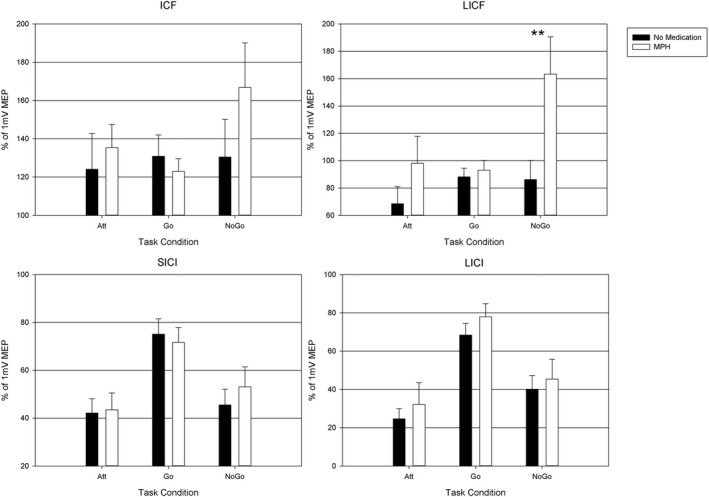
MPH and task condition effects on paired‐pulse TMS measures. MPH, methylphenidate, significant differences between MPH and no medication for nogo‐LICF is marked with ** for *p* < 0.001

Analyses revealed a MPH x condition interaction on the facilitatory TMS parameter LICF, due to the increased LICF facilitation under MPH medication under the nogo condition (*Z* = 3.24, *p* = 0.001, Table 1; Figure 2). Additionally, there was a strong task condition effect on the inhibitory TMS measures, as SICI and LICI were significantly increased in the nogo condition. Effects of MPH administration on the MEP amplitudes were already reported elsewhere (Buchmann et al., 2010). They found an MEP amplitude increase in the go condition for all ISI's, regardless if inhibitory or facilitating processes are examined and additionally for the referencing single pulse application. In the no go condition, only MEP amplitudes for the facilitatory motor processes (ISI13 and ISI 50) were increased by MPH and not the MEP to the single TMS pulse. This effect was leading to the increased LICF facilitation under MPH medication which is a result of our study.

**Table 1:** Variance analysis of MPH and condition effects on TMS parameter

Mean/*SD*					Statistical Effect	SICI		LICI		ICF		LICF	
Level	SICI	LICI	ICF	LICF		Z/*χ* ^2^	*p*	Z/*χ* ^2^	*p*	Z/*χ* ^2^	*p*	Z/*χ* ^2^	*p*
MPH	0.571/0.315	0.447/0.288	1.324/0.862	0.825/0.490	MPH	0.36	0.719	0.237	0.813	1.1	0.271	2.458	**0.014**
Baseline	0.622/0.342	0.529/0.477	1.499/0.666	1.269/0.881									
Cue	0.468/0.280	0.289/0.346	1.330/0.610	0.857/0.723	Condition	20.194	**<0.001**	35.68	**<0.001**	4.065	0.131	10.07	**0.007**
Go	0.762/0.243	0.716/0.293	1.301/0.390	0.887/0.280									
NoGo	0.537/0.316	0.437/0.388	1.571/0.810	1.338/1.081									
Cue*BL	0.457/0.337	0.255/0.295	1.271/1.039	0.702/0.704	MPH * Condition	0.467	0.762	1.267	0.531	2.867	0.239	16.47	**<0.001**
Cue*MPH	0.494/0.394	0.332/0.656	1.423/0.636	1.046/1.113									
Go*BL	0.766/0.340	0.685/0.339	1.364/0.633	0.865/0.326									
Go*MPH	0.772/0.312	0.768/0.368	1.219/0.382	0.933/0.387									
NoGo*BL	0.491/0.377	0.401/0.411	1.338/1.131	0.906/0.818									
NoGo*MPH	0.600/0.472	0.487/0.625	1.855/1.312	1.826/1.576									

MPH, methylphenidate; SICI, short‐interval intracortical inhibition; LICI, long‐interval intracortical inhibition; ICF, intracortical facilitation;LICF, long‐interval intracortical facilitation; nonparametric tests (Wilcoxon, Friedman) were used. Significant effects (*p* < 0.05) are marked in bold. For mean and standard derivation, see Figure 1.

### 3.2 Associations of ERP and task performance with TMS measurements under MPH medication

**Figure 3:**
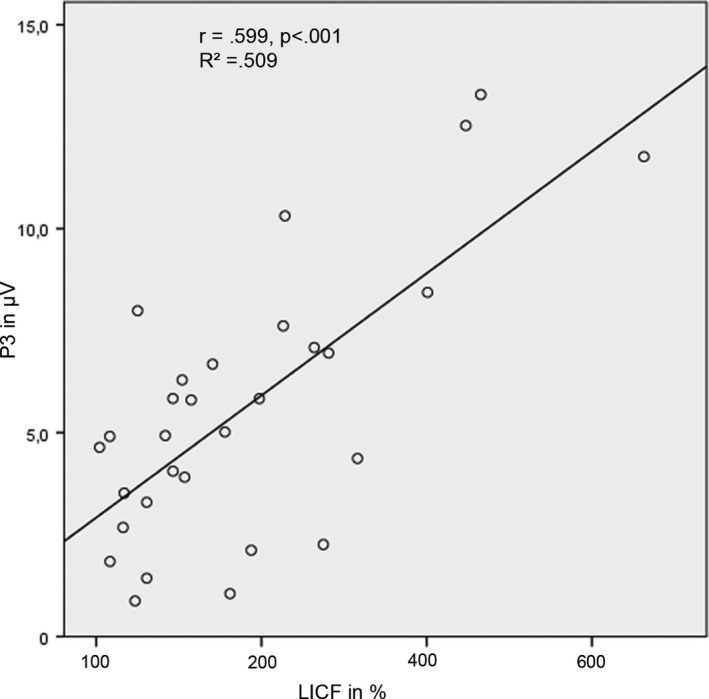
P3 peak amplitude correlation with motor cortex facilitation (LICF) under MPH medication in the nogo condition. LICF, long intracortical facilitation

Regarding the influence of MPH on the association between ERP and TMS measures, we found a significant positive correlation between LICF and P3 in the nogo condition under MPH. (*r* = 0.661, *p* < 0.001, Figure 3). We found no correlative association between any of the ERP or TMS measures with task performance.

### 3.3 Effects of MPH on ERP amplitudes and task performance

**Figure 4:**
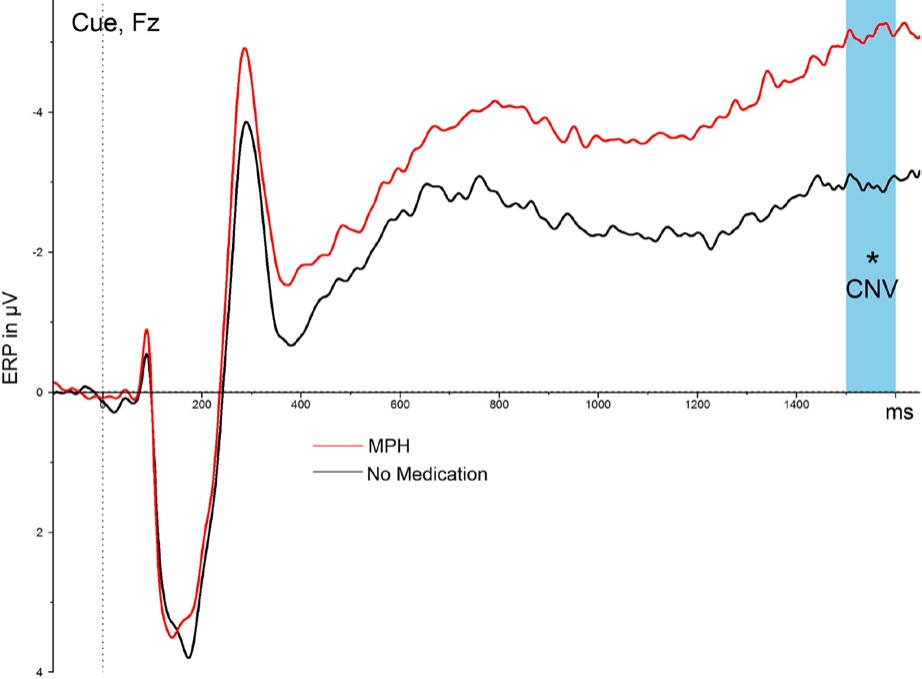
Effect of MPH on the contingent negative variation. MPH, methylphenidate; CNV, contingent negative variation measured from −100 ms to stimulus onset (blue window) *significant differences (*p* < 0.05) between MPH and no medication for CNV mean amplitude

Methylphenidate was affecting the attention during the cue condition: the CNV was more negatively pronounced under MPH, revealed by Wilcoxon test (Figure 4; Table 2). Furthermore, as expected, a paired *t* test revealed that MPH medication had an effect on the task reaction time and a Wilcoxon test revealed an MPH effect on the hit rate. The hit rate was increased and reaction was faster under MPH treatment (Table 2).

**Table 2:** CNV and task performance effects

ERP	Task	MPH	Mean	*SD*	*p* (2‐side)
CNV (µV/ms)	Cue	0	−2.978	6.288	**0.022**
		1	−5.208	3.735	
goRT (ms)	Go	0	370.458	86.477	**<0.00001**
		1	315.316	65.844	
Hit rate (%)		0	95.94	10.269	**0.003**
		1	99.69	0.710	

CNV, continuous negative variation; erp, event‐related potentials; goRT, reaction time of hits; hit rate, hits/*n* go trials; MPH, methylphenidate; *SD*, standard deviation. Bold *p* values < 0.05.

**Figure 5:**
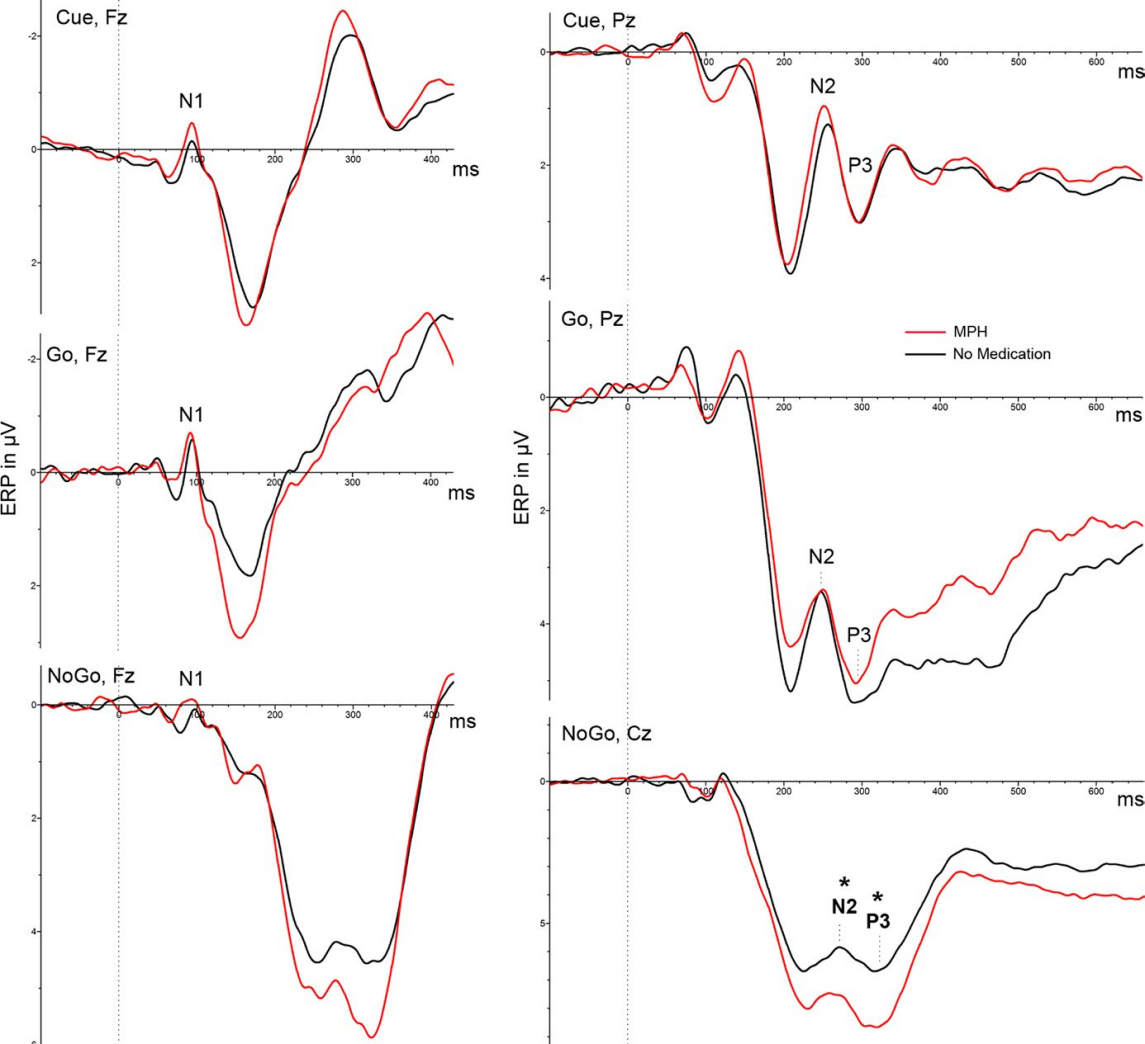
Effects of MPH and task condition on N1, N2, and P3 ERP. MPH, methylphenidate (red line), significant differences between MPH and No medication are marked with * for *p* < 0.05

**Figure 6:**
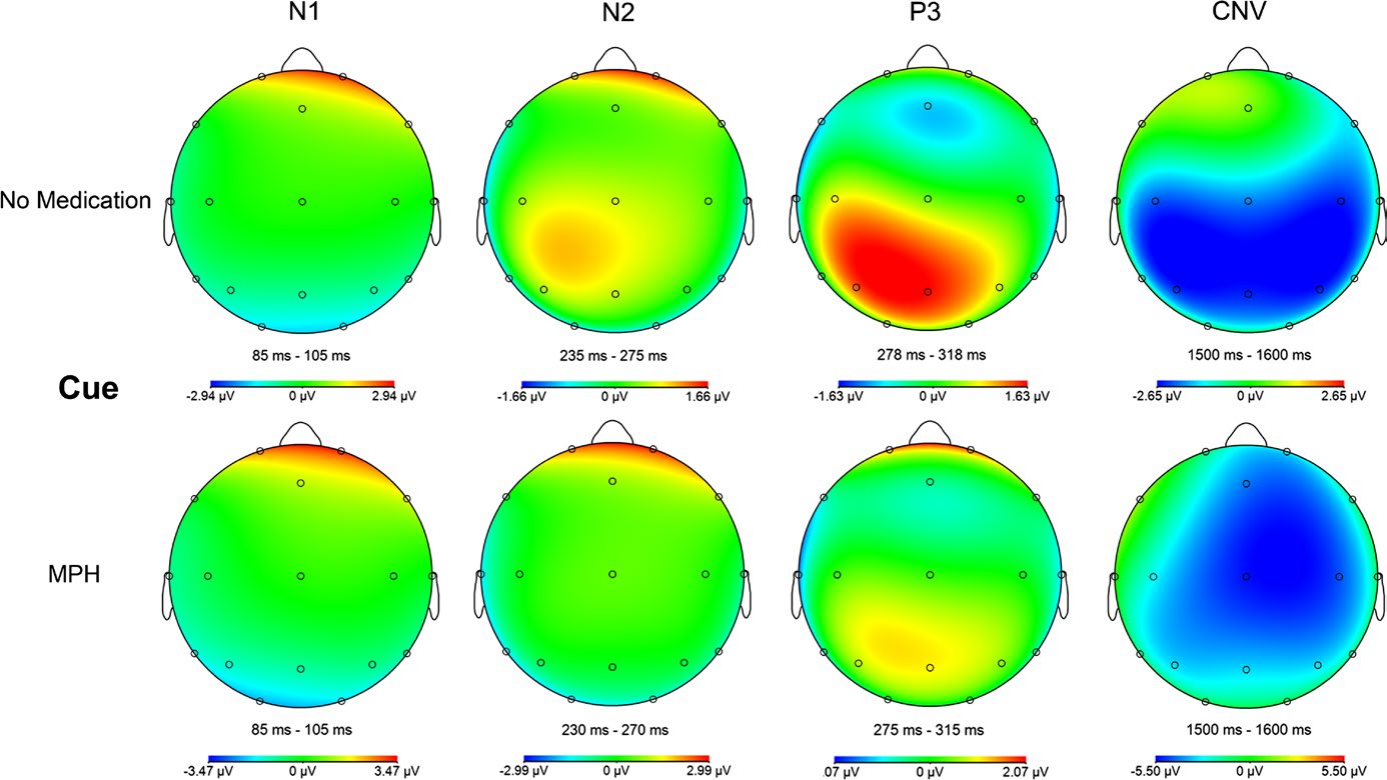
Topographic maps for cue task condition. The maps were based on ERP data without baseline correction in order to avoid topographic distortion, MPH, methylphenidate

**Figure 7:**
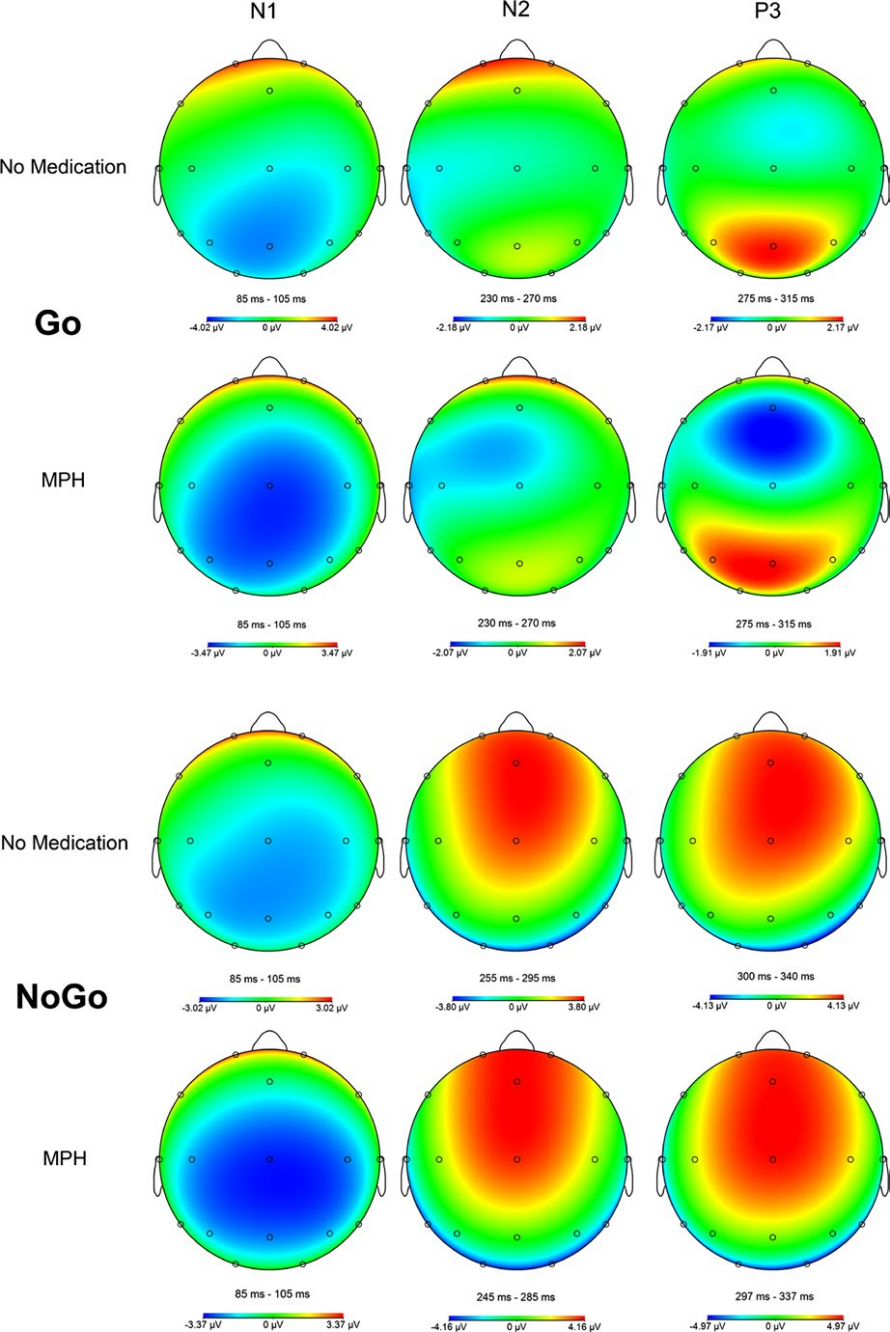
Topographic maps for go/nogo task condition. The maps were based on ERP data without baseline correction in order to avoid topographic distortion, MPH, methylphenidate

Furthermore, a repeated measures ANOVA revealed a main effect of task condition for all ERP (N1, N2, and P3), but not main effect of MPH. Additionally, we found an MPH‐by‐task condition interaction effect for N2 and P3 amplitude, but not for N1 (see Table 3). Post hoc *t* tests revealed that this MPH‐by‐task condition interaction was particularly driven by a more positively pronounced P3 and N2 peak amplitude under MPH compared to baseline for nogo (P3: *p* = 0.002, N2: *p* = 0.005; see Table 3; Figures 5, 6, 7).

**Table 3:** ANOVA of MPH and condition effects on ERP

Mean/*SD*				ANOVA effect	N1			N2			P3		
Level	N1	N2	P3		*df*	*F*	*p*	*df*	*F*	*p*	*df*	*F*	*p*
MPH	−0.771/1.129	3.037/2.248	6.593/2.537	MPH	1	1.311	0.261	1	0.505	0.483	1	1.651	0.209
Baseline	−0.582/1.050	2.750/2.114	6.111/2.470										
Cue	−0.597/1.121	0.481/1.804	3.932/1.768	Condition	1.625	5.207	**0.008**	1.394	40.55	**<0.001**	1.385	25.87	**<0.001**
Go	−1.024/1.404	2.604/2.102	6.397/2.749										
NoGo	−0.407/0.884	5.719/3.743	8.602/4.413										
Cue*BL	−0.475/1.157	0.655/1.892	3.849/1.951	MPH * Condition	1.521	0.451	0.587	1.618	6.729	**0.005**	1.442	8.956	**0**.**002**
Cue*MPH	−0.720/1.313	0.292/2.073	4.020/1.953										
Go*BL	−1.002/1.677	2.740/2.673	6.748/3.329										
Go*MPH	−1.046/1.487	2.467/2.550	6.133/2.974										
NoGo*BL	−0.268/1.193	4.856/3.972	7.735/4.614										
NoGo*MPH	−0.546/1.136	6.582/4.188	9.627/4.656										

*df*, degree of freedom; MPH, methylphenidate; *SD*, standard deviation. Bold *p* values < 0.05.

Furthermore, we found no gender differences and no gender by MPH interaction in the TMS, ERP, or performance measures.

### 3.4 Effects of MPH concentration and clearance on TMS excitatory effects, ERP amplitudes, and task performance

We tested the relation between MPH serum concentration and clearance, ERP and TMS effects and measures for performance affected by MPH via correlations of hit rate, reaction time, CNV, nogo‐N2, and nogo‐P3. We found no significant correlation neither with MPH serum level nor with MPH clearance.

## 4 DISCUSSION

This study investigated neuronal attention processes and motor cortex excitability and their pharmacological modulation by methylphenidate (MPH), measuring simultaneously event‐related potentials (ERP) and transcranial magnetic stimulation (TMS) during a go‐nogo task.

In our view, this study produced three remarkable findings. First, we observed an increased motor cortex facilitation under the nogo condition as a mismatch between MPH‐induced change in motor cortical excitability and the task demand, which can be interpreted as specific MPH effect on facilitatory motor circuits, here contradicting the task demands. Secondly, P3 was also increased in the nogo condition under MPH, indicating an enhanced evaluation of motor inhibition. Thirdly, we observed a strong correlation between P3 and LICF under the nogo condition, but only under MPH medication. Taken together, we interpret these results as a compensatory allocation of response control aimed at dimming the increased motor cortical facilitation, when contradicting the inhibitory task demands. Furthermore, the N2 was affected by MPH under the nogo condition, but—taking the time course of the ERP into account—we assume that the positively pronounced nogo N2 under MPH is due to the effect of the positive P3 complex. Regarding the N2 effect in our study, it should be stated that we were analyzing a posterior N2 effect. The N2 over frontal electrodes was essentially eliminated (Figure 5). A reason for this lack of anterior N2 in our study is probably the missing stimulus novelty, because this is known as the main driver for an anterior N2 (Folstein & Van Petten, 2008), whereas a posterior N2 is more elicited by familiar but unpredictable stimuli, especially by stimuli of simple shapes (Alho, Woods, Algazi, & Naatanen, 1992). Finally, MPH enhances in particular attentional and response readiness processes vectored by expectations, as indicated by an increased CNV. We found no effect of MPH on early target related attentional processes (N1) and no effect on response execution control (go‐P3). With regard to the TMS measures, the change of MEP response to paired TMS pulse protocols and its relation to the single pulse MEP response was analyzed. We found a main effect of MPH and a MPH x condition interaction on the LICF, which showed increased facilitation under MPH medication for the nogo condition. In general, we found no correlation of MPH serum level with any of the experimental measures. At the performance level, we found faster reaction times and improved hit rates under MPH in the go condition, confirming the well‐known pro‐vigilant effect of MPH in healthy subjects (e.g., Bagot & Kaminer, 2014; Franke, Bagusat, Rust, Engel, & Lieb, 2014).

In our repeated measures design, the test condition under MPH administration was always following the baseline condition after a period of 6 weeks. Nevertheless, it can be assumed that carryover effects on performance and physiological recordings should be small. Training effects are expectable, when the task is complex and the inter‐test interval is short (min to hr; Buehner, Ziegler, Bohnes, & Lauterbach, 2006), which was not the case here. Moreover, ERPs and performance measures associated with the go/no go paradigm were reported to be reliable, for short‐term (Fallgatter et al., 2001) and long‐term (Brunner et al., 2013; Fallgatter, Aranda, Bartsch, & Herrmann, 2002) test/retest intervals.

Compensatory associations between attentional control and motor cortical activity have been described before, but only within the context of inhibitory intracortical connections. Hoegl et al. (2011) reported a significant negative correlation between short intercortical inhibition (SICI) and the P3 component for the nogo condition under MPH medication in healthy adults. Lower motor cortical inhibition measured by SICI at 120–350 ms post nogo stimulus was accompanied by an increased P3. A correlation between the nogo‐P3 and SICI at 500 ms after nogo stimulus was described by Heinrich et al. (2014) in children with ADHD. Higher response control reflected by higher P3 was followed by a better motor cortex inhibition. This correlation was not found in the age‐matched control group. The authors interpreted these results as a need for higher terminal response control in order to facilitate correct nogo response, signified by higher P3 amplitude when lower inhibitory effects in the motor cortex are measured. As a consequence, it seems to be reasonable to conclude for the P3‐LICF correlation in this study that complementary processes of increased attentional control are needed to deal with the mismatch between the MPH‐induced facilitation and nogo task condition calling for inhibition.

In earlier studies (Fallgatter & Strik, 1999; Pfefferbaum, Ford, Weller, & Kopell, 1985), the nogo‐N2‐P3 component was interpreted as a marker of motor inhibition. In more recent studies, it was interpreted differently because nogo‐P3 appeared too late in order to prove an online process of inhibition (Falkenstein et al., 1999). Nogo‐P3 waves rather seem to represent the evaluation of inhibition (Beste, Dziobek, Hielscher, Willemssen, & Falkenstein, 2009; Bruin, Wijers, & van Staveren, 2001) and/or the termination of motor activation (Kopp, Mattler, Goertz, & Rist, 1996). Moreover, differences of N2 and P3 amplitudes between go and nogo trials may possibly be explained by the smaller numbers of nogo trials in many studies as suggested by Shahaf, Fisher, Aharon‐Peretz, and Pratt (2015). Latter authors attributed the N2‐P3 complex to attention processes preceding response selection which are enforced by stimulus rarity, irrespective of whether response execution or inhibition was required. Nieuwenhuis, Yeung, Wildenberg, and Ridderinkhof (2003) concluded the same for the N2.

We found the P3 to be increased for the nogo condition under MPH medication, while no significant changes in P3 occurred in the go and cue condition. This result seems to signify MPH effects on context updating, but not on response execution processes or attentional allocation. Our results indicate that resource allocation for context updating processes was enhanced by MPH only if the inhibition of a response was required. In contrast to our findings, Hoegl et al. found the P3 amplitude to be significantly increased under MPH in the go, but not in the nogo condition (Hoegl et al., 2011), possibly due to increased task demand on response control because of used left hand and right hand go events.

We found a significant MPH effect as well as an MPH x condition interaction on the motor cortical facilitation; LICF was increased under MPH medication in the nogo condition, while the motor cortex inhibition (SICI or LICI) was not affected by MPH. This kind of increased motor cortex facilitation under MPH was reported in many studies with healthy adults, while inconsistent results have been found for intracortical inhibition. Some researchers, such as Gilbert et al. (2006) and Ilic et al. (2003) found a MPH‐induced decrease of inhibition (SICI; Gilbert et al., 2006; Ilic et al., 2003), while other reported either an increased inhibition (Kirschner et al., 2003) or no significant MPH effects on SICI (Moll et al., 2003). Nevertheless, in all studies reported here the short intracortical facilitation (ICF) was increased by MPH.

With regard to the intended direct comparisons of MPH effects on motor cortical facilitation measured by TMS under different task demands, it is worth to recognize that the target hand muscles are activated in correctly responded go trials, while the hand muscles remain at rest in correctly inhibited no go trials. In fact, voluntary contractions of the target muscle are leading to an increased facilitation of response to brain stimulation, which probably is caused by two reasons, focusing of attention onto particular hand and second a rise in excitability of pre‐innervated motor pathways (Hess, Mills, & Murray, 1986). This effect was leading to the increased MEPs (up to three times higher) in the go condition in general, as reported before (Buchmann et al., 2010).

We observed an increase of the CNV by MPH. The late wave of the CNV, as measured here, is assumed to be a mixture of expectancy related attention processes and response readiness motor preparation (Brunia & van Boxtel, 2001). The MPH‐induced increase of the stimulus and response anticipation measured by the CNV (and an additionally improved response performance) was already reported by Linssen et al. (2011) and is assumed to be induced by the MPH related enhancement of dopaminergic activity in frontocortical circuits (Linssen et al., 2011).

Furthermore, we observed no MPH‐induced difference in N1, indicating that early attentional orienting and stimulus evaluation processes were not affected by dopaminergic modulation. This finding corresponds to the results of Hoegl et al. (2011) who also found no effect of a single dose of MPH (20 mg) on either N1 or N2. Overtoom et al (2009) and Ohlmeier et al. (2007), however, found no effects using a low dose (0,4 mg/kg) of MPH, but found changes in the N1 and P3 in a stop signal task using a higher dosage (0,6 mg/kg). In the study by Overtoom et al. (2009), MPH was reported to restore the formerly absent N1 and to reduce the P3 in adult subjects with ADHD (Overtoom et al., 2009). Regarding the N1, it seems that MPH differently modulates the impaired sensory gating mechanism in subjects with ADHD compared to healthy individuals. This is in line with findings showing that lower levels of dopamine in unmedicated ADHD in comparison to healthy subjects (Volkow et al., 2007) could be responsible for this ADHD‐specific effect of MPH on the N1 ERP.

## 5 CONCLUSIONS

The ERP data in this study showed that expectancy related attention processes and response readiness motor preparation were increased by MPH. This enhancement regarded increased CNV mean amplitudes under MPH which may be interpreted as the neuronal correlate of increased vigilance and increased task performance. Later context updating (P3) was enhanced in the nogo condition, but only when needed by increased motor cortex facilitation (LICF) that needs to be compensated for. We interpret this context updating as a compensatory attentional response control mechanism in case of an over facilitated motor cortex.

Our results may be limited by the rather easy task condition. Therefore, we may not have been able to find any possible MPH‐induced changes in commission errors. The commission error rate in our study was in general too low for a statistical analysis. We did not test dosage‐dependent effects of MPH; therefore, it is still unclear whether lower MPH dosage could have effects on the interplay between motor cortex facilitation and attention processes in healthy subjects. Further research should highlight this motor‐attentional association in the context of ADHD. It remains an open question whether the presented results could be replicated in patients with disturbed response control. We assume that further studies with simultaneous measurement of attentional control and motor cortex excitability could expand our understanding of cognitive processes which control motor execution.

## CONFLICT OF INTEREST

The authors declare no conflict of interest.
